# Situation Actuelle de la Péripneumonie Contagieuse Bovine en République Centrafricaine

**DOI:** 10.48327/mtsibulletin.2021.100

**Published:** 2021-05-17

**Authors:** P. Ngounda, L. Manso-Silván, F. Thiaucourt

**Affiliations:** 1 Laboratoire central vétérinaire (LACEVET), Ministère de l'élevage et de la santé animale (MESA) BP 786 Bangui, République centrafricaine; 2 CIRAD, UMR ASTRE, F-34398 Montpellier, France; 3 INRAE, UMR1309 ASTRE, F-34398 Montpellier, France

**Keywords:** Péripneumonie contagieuse bovine, *Mycoplasma mycoides mycoides,*, Typage moléculaire, Tétracyclines, République centrafricaine, Afrique subsaharienne, Contagious Bovine Pleuropneumonia, *Mycoplasma mycoides mycoides*, Central African Republic, Molecular typing, Tetracyclines, Sub-Saharan Africa

## Abstract

**Matériel et méthodes:**

La situation de la péripneumonie contagieuse bovine en République centrafricaine (RCA) a été analysée par une enquête sérologique sur 776 sérums, à l'aide de la technique cELISA. La prévalence de la maladie a été estimée à 12,5% quelle que soit la région.

**Résultats:**

Une différence significative a été observée entre les zébus et la race taurine, probablement associée au mode d'élevage, favorisant plus ou moins les contacts. La présence de la maladie dans les trois régions d'élevage a également été confirmée par l'isolement de souches de *Mycoplasma mycoides mycoides* lors de foyers de 2019. Ces souches sont toujours sensibles aux tétracyclines. Les trois souches dont le génome a été séquencé étaient strictement identiques. Replacés dans un arbre phylogénétique, ces génomes sont proches de ceux des souches isolées précédemment en RCA, ce qui montre que la maladie y est enzootique.

**Conclusion:**

L'amélioration de la situation ne pourra venir que d'une modification des stratégies de lutte et d'un retour au calme sur le terrain afin de pouvoir les appliquer.

## Abstract (en)

**Aims:**

The aim of this study was to analyse the current situation of Contagious Bovine Pleuropneumonia (CBPP) in the Central African Republic (CAR) by seroprevalence analysis, as well as isolation and characterization of strains of the etiologic agent, *Mycoplasma mycoides mycoides* (Mmm), circulating in livestock breeding regions.

**Material and methods:**

The strains obtained were subjected to whole genome sequencing by Illumina technology and genotyped using the eMLST technique based on 62 genes of the Mmm core genome. Their sensitivity to tetracycline was assessed by determination of the minimum inhibitory concentration (MIC) on agar. A seroprevalence analysis by competitive ELISA was conducted in livestock breeding regions (West, Centre and East CAR), including both zebu and taurine cattle breeds, and both males and females.

**Results:**

The three strains isolated in the three regions of the CAR shared exactly the same genomic sequence. Phylogenetic analysis showed that they were closely related to a strain isolated in the CAR in 1991, also sequenced in this study, and clustered with Mmm strains originating from East and Central Africa. The recent isolates presented increased MIC values, though they were still sensitive to tetracycline. The global CBPP prevalence in the CAR was estimated at 12.5% with no significant differences observed between cattle breeding regions, nor between males and females. However, a significantly higher prevalence was observed in zebu compared to taurine cattle, most likely in relation to their herding system based on cattle transhumance and nomadic pastoralism.

**Conclusion:**

CBPP is enzootic in the CAR in spite of control campaigns based on use of the live T1 vaccines, which have shown little efficacy due to poor implementation in the field. New strategies combining controlled use of antibiotics and inactivated vaccines, with increased thermostability, should be well received by livestock keepers and allow a better control of CBPP in the region. The fact that the recent Mmm isolates are still resistant to tetracycles is encouraging.

## Introduction

La République centrafricaine (RCA) est un pays où l'élevage des bovins est relativement récent. En effet, c'est à partir des années 1920 que du bétail a été introduit par des Peuls Mbororo par l'ouest, dans ce qui était autrefois appelé l'Oubangui-Chari [11]. La principale limitation à cet élevage a surtout été la trypanosomose, en raison de la présence de glossines dans les zones forestières. Malgré cela, le cheptel bovin n'a cessé de croître en RCA et le cheptel, estimé à 700000 vers 1967, serait maintenant d'environ 4 à 5 millions de têtes. Depuis l'ouest du pays, l'élevage s'est étendu à la fois vers l'est et le sud pour atteindre la frontière avec la République démocratique du Congo (RDC) et des zones frontalières avec le Sud Soudan, alors que celles-ci étaient considérées, jusqu'ici, comme des réserves de faune sauvage. L'augmentation du cheptel a été grandement facilitée par la diffusion des médicaments vétérinaires, qui a compensé en partie la disparition de l'encadrement vétérinaire [15].

**Figure 1:**
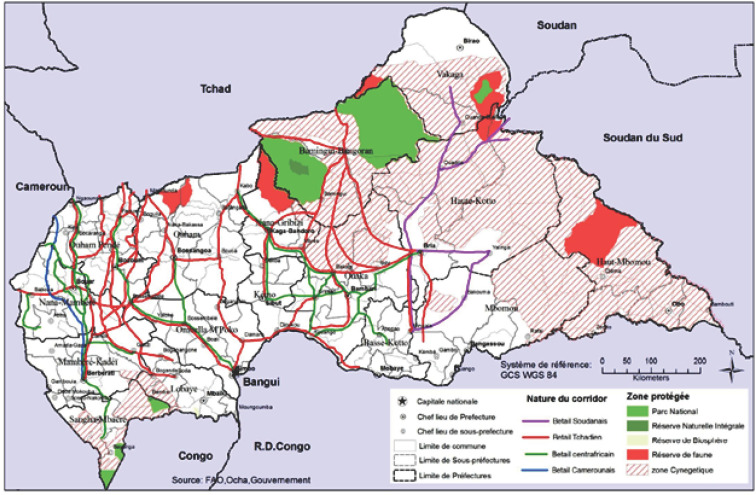
Couloirs pastoraux en RCA avant 2015 Transit corridors in the CAR before 2015 Source: https://fscluster.org/central-african-republic/document/fsc-car-transhumance-corridors-pastoraux Avant 2015, des couloirs pastoraux permettaient au bétail de circuler, principalement du nord vers le sud afin d'alimenter les marchés au bétail

**Figure 2:**
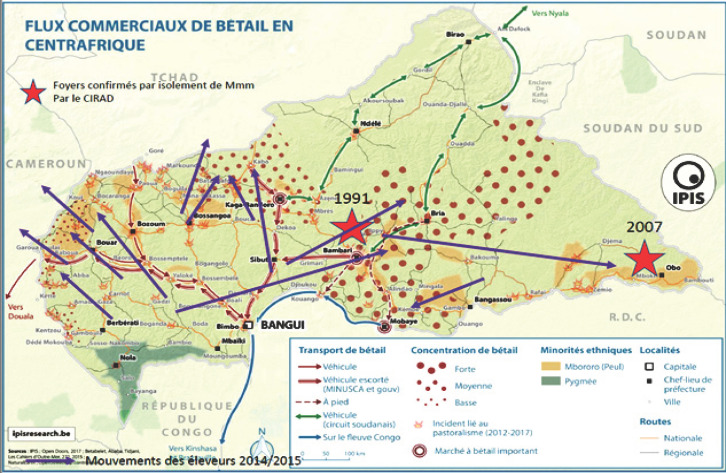
Mouvements du bétail et troubles civils en RCA Livestock movements and civil unrest in the CAR Adapté de https://ipisresearch.be/wp-content/uploads/2017/08/201708_caf_pastoralisme_A1.png Les mouvements d'éleveurs de 2014/2015 sont figurés par des flèches violettes. Ces mouvements ont entraîné une modification des densités animales et une raréfaction des bovins dans un rayon d'au moins 200km autour de la capitale Bangui. Les deux foyers de PPCB qui avaient été confirmés par le passé, sont figurés par des étoiles rouges, à Bambari en 1991 et dans la région d'Obo en 2007

Ce qui caractérise l'élevage en RCA, c'est l'intensité des mouvements du bétail, facteur majeur de risque de transmission de maladies comme la péripneumonie contagieuse bovine (PPCB). Avant 2015, les mouvements de bétail se faisaient à travers des corridors, en particulier pour les flux commerciaux en provenance des pays voisins au nord et à l'ouest (Soudan, Tchad, Cameroun) ou bien pour des mouvements de transhumance intra RCA ou avec le Tchad (Fig. 1). Les troubles civils qui ont affecté la RCA en 2015 ont fortement perturbé l'élevage. Les éleveurs ont quitté certaines régions où ils s'étaient installés, dans l'ouest du pays, et se sont dispersés largement (Fig. 2). Les mouvements commerciaux continuent, ne serait-ce que pour approvisionner la capitale, Bangui, ou bien pour des marchés d'exportation plus au sud vers la RDC. L'insécurité persiste cependant sur les couloirs de mouvements d'animaux et le vol de bétail est devenu fréquent, ce qui rend encore plus difficile le contrôle des mouvements de bovins.

La PPCB n'est pas une maladie nouvelle en RCA. Elle était déjà connue au Tchad voisin, en 1924 [10] et une première incursion a été décrite en 1958 par Desrotour et Itard [2]. Ce premier foyer en RCA y a alors été éradiqué grâce à un contrôle strict des mouvements de bétail et l'utilisation d'une souche vaccinale assez virulente (souche T3 de Piercy) qui, injectée dans le mufle, induisait la mortalité des individus contaminés, mais protégeait les autres. L'extension de l'élevage bovin et des mouvements d'animaux n'ont pas permis de garder le statut indemne de PPCB à la RCA et d'autres foyers furent officiellement confirmés par l'envoi de prélèvements à l'Institut d'élevage et de médecine vétérinaire des pays tropicaux en 1991, puis au CIRAD en 2007, provenant de Bambari et MBoki respectivement (Fig. 2). La RCA a déclaré régulièrement la présence de la PPCB sur son territoire auprès de l'Organisation mondiale de la santé animale (OIE).

**Figure 3:**
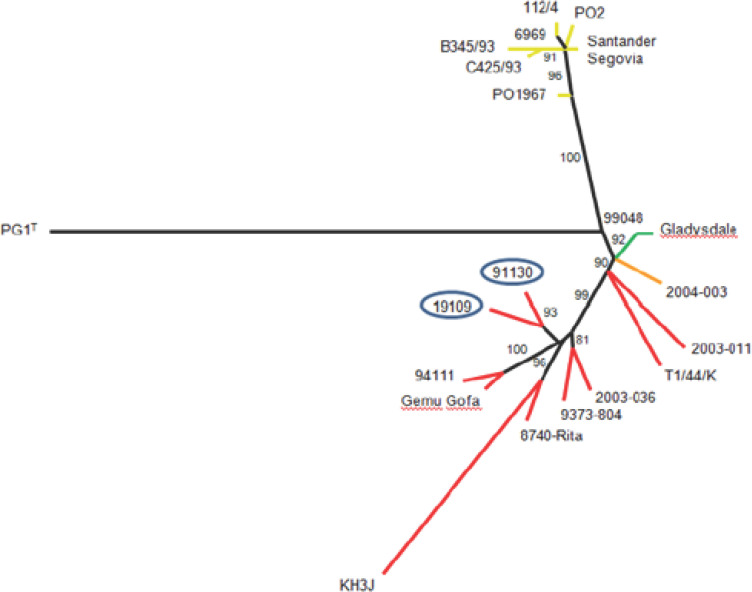
Analyse phylogénétique des souches isolées en RCA par la technique de « eMLST » Phylogenetic analysis of strains isolated in the CAR by the “eMLST” technique Les positions polymorphes de chacune des 23 souches, présentes sur les 62 gènes retenus pour l'analyse, ont été concaténées pour générer un fichier multifasta de 23 lignes de 145 caractères (nombre total de SNPs). Les séquences ont été alignées et analysées en maximum de vraisemblance avec 1000 bootstraps. Les valeurs aux embranchements représentent le % de bootstrap. Les souches d'origine sub-saharienne sont représentées à l'extrémité de branches rouges (orange pour Afrique Australe, vert pour Inde et Australie, jaune pour l'Europe). Les deux souches de RCA sont entourées. Elles dérivent d'un ancêtre commun et d'un embranchement à partir duquel les souches d'Afrique de l'Est et du Centre sont également issues (Dupuy et al 2012)

Des campagnes de vaccination sont régulièrement organisées, à travers une coordination de l'Organisation des Nations unies pour l'alimentation et l'agriculture (FAO) et avec l'aide de certaines organisations non gouvernementales (ONG) dans la mesure où la situation sécuritaire ne permet pas aux vétérinaires du ministère de l'élevage et de la santé animale d'intervenir sur tout le territoire. En 2016, une campagne de vaccination contre la PPCB a été réalisée sur financement de l'Union européenne, organisée par la FAO et l'Agence nationale de développement de l'élevage (ANDE) avec l'aide d'ONG (Triangle, ACTED): 260728 animaux ont été vaccinés pour un objectif de 1500000 têtes prévues, soit un taux de réussite de 17,38%. En 2017, pour un objectif de vaccination de 856000 animaux, 540856 têtes ont été vaccinées soit un taux de vaccination de 69,18% [5]. Ces deux campagnes de vaccination, effectuées en 2016 et 2017, qui n'ont cependant pas couvert toutes les localités des trois régions du pays, ont été réalisées avec les vaccins de souche T1-44 certifiés par le laboratoire panafricain de contrôle des vaccins vétérinaires (AU-PANVAC).

Malgré cela, des lésions typiques de PPCB ont été observées à l'abattoir de Bangui, et une étude préliminaire avait estimé la prévalence sérologique à 7,6%, indépendamment des régions d'élevage, race, sexe et âge [9].

L'objectif de cette étude a d'abord été de confirmer l'étiologie des lésions par l'isolement de *Mycoplasma mycoides mycoides* (Mmm) l'agent de la PPCB, de typer génétiquement les souches obtenues, puis d'estimer leur sensibilité aux tétracyclines. Enfin, une enquête sérologique a été réalisée afin de déterminer la prévalence de la PPCB dans les zones où est pratiqué l'élevage.

## Matériel et Méthode

### Typage des souches Mmm de RCA

Trois souches ont été isolées en 2019 à partir d'échantillons de lésions pulmonaires en provenance de l'est, du centre et de l'ouest du pays après ensemencement de milieu gélosé « Hayflick modifié » [13] additionné de rifampicine (10 mg·l-1) et d'amphotéricine, (0,1 g·l-1). Après clonage pour assurer la pureté des souches, celles-ci ont été cultivées dans 5 ml de milieu Hayflick pendant 48 heures. Les culots bactériens ont été concentrés par centrifugation à 12000 g pendant 30 minutes puis repris dans 200 μl d'eau. Les ADN ont été extraits avec le kit Qiagen « DNeasy Blood & Tissue Kit » (ID: 69504), puis envoyés pour séquençage Illumina (Macrogen République de Corée: Librairie Illumina TruSeq DNA Nano, Illumina HiSeqX 2x250bp).

Les génomes de ces trois souches ont été comparés en réalisant des assemblages des séquences illumina sur le génome complet de la souche Mmm « gladysdale » (CP002107), en utilisant le logiciel Seqman NGen (DNAStar, Lasergene, V14.1.0). La liste des positions uniques polymorphiques fixées (SNPs), % > 75, a été exportée pour chaque souche et comparées entre elles.

Une analyse phylogénétique a été réalisée par la technique « enlarged Multi-Locus Sequence Typing » (eMLST), qui inclut 62 gènes appartenant au « core genome » de Mmm [3]. Vingt-trois souches ont été analysées, les 21 premières correspondant à la publication initiale et deux autres en provenance de RCA, l'une isolée en 1991 (91130) et une des plus récentes (19109). Pour ce faire, un assemblage a d'abord été réalisé pour chaque souche avec une séquence concaténée des 62 gènes avec des zones flanquantes (SeqMan NGen). Les séquences consensus ont été exportées, puis ensuite assemblées dans le logiciel SeqMan Pro (V14.1.0). L'assemblage consensus a été débarrassé des séquences flanquantes et de toutes les positions invariables entre 2 SNP pour ne plus conserver que les positions variables. Celles-ci ont été exportées dans un fichier multifasta.

Ce fichier multifasta a été utilisé pour réaliser une analyse phylogénétique (MEGA6) [12]. Un alignement a été d'abord généré (Clustal W), puis analysé par la méthode de maximum de vraisemblance: 1000 bootstraps, gTR model, gamma distribution, arbre initial généré par « Neighbor Joining ». L'arbre obtenu a été exporté en format Newick pour une observation avec le logiciel Figtree (https://github.com/rambaut/figtree/releases).

### Établissement de la concentration minimale inhibitrice des souches Mmm de RCA

Afin de vérifier si les souches récentes de Mmm isolées en RCA étaient toujours sensibles à la tétracycline, une analyse a été réalisée selon le protocole du CIRAD en vigueur. Brièvement, les trois souches isolées en 2019 ainsi que la souche isolée en 1991 ont été mises en culture en dilutions successives au 1/10. Après 48 heures d'incubation, l'avant dernier tube présentant un trouble a été sélectionné pour réaliser une dilution à 10-5. Cinq microlitres de cette dilution ont été déposés sur des géloses contenant des concentrations variables de chlorhydrate de tétracycline (Sigma Aldrich T7660), de 2 μg à 0,06 μg·l-1 ainsi qu'une gélose témoin ne contenant pas d'antibiotique.

Les géloses ont été mises à incuber à 37°C et la lecture effectuée après 5 jours d'incubation. La lecture a été considérée comme conforme pour une souche, quand au moins 10 colonies bien séparées ont été observées dans la gélose témoin sans antibiotique. La concentration minimale inhibitrice (CMI) a correspondu à la concentration minimale d'antibiotique ayant significativement inhibé la croissance des colonies pour cette souche.

### Séroprévalence de la PPCB en RCA

Une estimation de la prévalence de la PPCB a été obtenue par la sérologie. Des sérums ont été récoltés dans les trois régions (ouest, centre et est) où est pratiqué l'élevage bovin en RCA. Les élevages de type sédentaire et transhumant ont été ciblés par l'étude. Dans les zones d'élevage (sédentaire et transhumant) et les marchés de bétail, le choix des élevages dans une zone a été fait au hasard avec l'appui des chefs de secteurs et des postes vétérinaires sur la concentration des troupeaux dans la zone. Le choix des animaux prélevés a été fait également au hasard et le taux de prélèvement dans les troupeaux a varié entre 10 et 20% en fonction de l'effectif et sur accord de l'éleveur respectivement pour les troupeaux de plus de 50 têtes et pour les troupeaux de moins de 50 têtes. Sept cent soixante-seize sérums ont été testés par la technique cELISA [6] qui est un test prescrit par l'OIE (kit IDEXX ref P05410-10). Les différences de pourcentages de séropositivité ont été analysés par la méthode des écarts réduits.

## Résultats

### Typage des souches de Mmm

Les trois souches isolées de trois régions de RCA possédaient exactement la même séquence génomique, car aucun SNP spécifique de souche n'a pu être identifié sur les 303 SNP observés par rapport au génome de la souche gladysdale. C'est pour cela que l'analyse phylogénétique n'a été réalisée que pour l'une d'entre elles (19109).

L'analyse eMLST sur 62 gènes et 23 souches a permis d'identifier 145 SNP, soit 6 de plus que dans l'étude précédente qui ne comprenait pas de souche en provenance de RCA. L'arbre généré en maximum de vraisemblance a une topologie tout à fait similaire à celui généré en 2012 (Fig. 3).

Une branche supplémentaire est apparue, à partir de la même racine que les branches portant les séquences du Rwanda (94111) et d'Ethiopie (Gemu gofa) d'un côté et du Cameroun (8740) et du Soudan (KH3J) de l'autre côté. Les deux séquences provenant des souches de RCA se trouvent sur deux sous-branches séparées. Cela montre que 19109 ne dérive pas directement de la souche isolée en 1991 mais est bien issue d'un ancêtre commun.

### CMI des souches Mmm de RCA

La croissance de la souche la plus ancienne a été inhibée à la concentration la plus basse, 0,06 μg·l^-1^. Pour les trois autres, la CMI a été de 0,5 μg·l^-1^.

### Séroprévalence de la PPCB

Les pourcentages de positifs observés sont présentés dans le tableau 1. Lorsqu'on considère la zone de provenance des animaux, il ressort de ce tableau qu'il n'y a pas de différence significative entre la prévalence des régions de l'Ouest et du Centre. La prévalence est globalement uniforme sur tout le territoire et voisine de 12,5%. De même, aucune différence significative n'a été observée en fonction du sexe, les mâles aussi bien que les femelles ont été touchés par la PPCB. Par contre, des prévalences significativement différentes ont été observées entre les races taurines et les races zébus (M'bororo et goudali).

**Tableau I:** Pourcentages de sérologies positives en cELISA PPCB, selon la région, le sexe ou la race bovine Percentages of positive cELISA CBPP serologies, by region, sex or bovine breed

	DRO	DRE	DRC	Mbororo	Goudali	Taurine	Mâles	Femelles
**Nb**	223	288	265	416	285	75	448	328
**Positifs**	21	41	35	66	29	2	61	36
**%**	9,4	14,2	13,2	15,9	10,2	2,7	13,6	11,0

* Les pourcentages sur une même ligne ne sont pas statistiquement différents. Seul le pourcentage de positifs chez la race taurine diffère des pourcentages des races de zébu. Cela est sans doute à mettre en relation avec des modes d'élevage différents. Direction régionale de l'Est, DRE; Direction régionale du centre DRC; Direction régionale de l'Ouest, DRO.

## Discussion

La stricte identité des génomes des trois souches Mmm, isolées en RCA en 2019 en provenance de trois régions différentes, montre qu'une souche particulièrement virulente a diffusé récemment en RCA sur l'ensemble du territoire. Cela peut sans doute être mis en relation avec les mouvements non contrôlés de bétail, qui ont survenu notamment lors des crises de 2014/2015. Il ne s'agit pas d'une souche « importée » puisque le génome des souches de 2019 est proche du génome d'une souche isolée en RCA en 1991. Cette persistance de la PPCB en RCA peut facilement s'expliquer par une couverture vaccinale à la fois partielle et variable d'une année sur l'autre, qui ne permet pas de contrôler la PPCB. En effet, il est admis que, pour contrôler efficacement la PPCB par la vaccination avec les souches type T1, il faut cibler 100% de la population sensible et réaliser au moins trois campagnes annuelles successives pour établir une immunité de groupe. Pour que les campagnes de vaccination soient efficaces, il faut également s'assurer que les flacons de vaccin proviennent d'un lot conforme au départ. Une certification du lot par le AU-PANVAC permet de s'en assurer et c'était le cas en RCA. Mais il faut aussi qu'ils soient conservés correctement jusqu'au pied de l'animal et soient ensuite administrés selon les règles de l'art. En pratique, l'expérience montre que, sur le terrain, ce n'est pas toujours le cas. Dans ces conditions, l'immunité acquise par les troupeaux peut être largement inférieure à ce qu'on serait en droit d'attendre d'une campagne correctement réalisée.

La CMI pour la tétracycline des souches de 2019 a été augmentée, mais cette CMI correspond toujours à une sensibilité des souches par rapport à cet antibiotique, comme cela avait été montré en 2005 [1]. Cela peut surprendre étant donné la large utilisation, notamment des tétracyclines longue action, en Afrique. En pratique, in vitro, il est relativement difficile d'obtenir des souches Mmm résistantes à la tétracycline, contrairement à ce qui peut être obtenu avec les aminosides, par exemple [7]. Une situation similaire pourrait éventuellement exister in vivo, alors que la posologie habituelle pour des tétracyclines longue action est de l'ordre de 20 mg·kg-1 (20 μg·g-1), soit largement au-dessus de la CMI évaluée lors de cette étude.

La prévalence globale de la PPCB en RCA est de 12,5%. Cette prévalence est supérieure à celle de la campagne sérologique de 2016 qui était de 7,7% [9], cependant sans différence significative, et cela montre que la PPCB est bien enzootique en RCA. Par ailleurs, nous n'avons pas observé de différence significative entre les régions. Les mouvements désorganisés de bétail, le regroupement des éleveurs en certaines zones et l'absence d'application des dispositions sanitaires relatives à la transhumance, pendant la période de la crise, pourraient expliquer ces résultats. Il n'y a pas eu non plus de différence significative de prévalence observée entre les femelles et les mâles, ce qui est en accord avec ce qui est connu de la PPCB et de ses facteurs de risque.

Des prévalences significativement différentes ont été observées entre les races taurines et les races zébus (M'bororo et goudali). Les conditions d'élevage pourraient expliquer ces différences. Cela corrobore les résultats de Lefèvre selon lesquels l'atteinte fréquente de zébu est liée au mode d'élevage [8]. Les animaux de race taurine sont élevés généralement en sédentarité, à proximité des exploitations agricoles et utilisés en culture attelée avec des déplacements très limités autour des parcelles agricoles. Cependant, les animaux de races M'bororo et goudali effectuent de longs déplacements pendant les périodes de transhumance et sont en contact avec les autres animaux de la transhumance transfrontalière autour des points d'eau ou dans les pâturages.

## Conclusion et Perspectives

Il ressort de cette étude que la PPCB est toujours enzootique sur l'ensemble du territoire de RCA avec une prévalence de l'ordre de 12,5%. Le typage génétique des souches isolées récemment montre que ce sont des souches endogènes qui continuent de circuler. Ces résultats montrent clairement que les campagnes de lutte contre la PPCB n'ont pas permis de contrôler cette maladie. En fait, la PPCB n'a été effectivement contrôlée que pendant les campagnes de lutte conjointes contre la peste bovine et la PPCB [14]. Une fois la peste bovine éradiquée, les financements internationaux se sont taris et les moyens ont manqué pour contrôler efficacement la PPCB, non seulement en RCA mais sur l'ensemble des pays africains de la zone intertropicale.

Les troubles civils qui continuent de sévir en RCA n'augurent rien de bon pour le contrôle de la PPCB et les éleveurs vont encore payer un lourd tribut à cette maladie.

Il est évident que les stratégies de lutte actuelles, basées uniquement sur l'utilisation des vaccins vivants de type T1, ne sont pas efficaces. Cela n'est pas dû au vaccin lui-même, mais plutôt aux conditions de son utilisation sur le terrain. Une amélioration significative de la situation ne pourra venir que d'un changement de paradigme pour les stratégies de lutte [4]. L'utilisation de vaccins de type « inactivé » pourrait résoudre les difficultés liées aux ruptures de la chaîne du froid. Par ailleurs, il conviendrait de promouvoir des traitements antibiotiques bien conduits, lors de la détection de foyers. Cela offrirait de multiples avantages: inspirer confiance aux éleveurs qui ne rechigneraient plus à déclarer des foyers et réduire l'excrétion de Mmm par les animaux malades et donc diminuer le risque pour les animaux ou élevages voisins. A ce titre, le fait que les souches récentes de Mmm soient encore sensibles aux tétracyclines est un signe encourageant.

## Conflits D'intérêts

Les auteurs ne déclarent aucun conflit d'intérêt.
